# Supplementary material for: Cloud BioLinux: pre-configured and on-demand bioinformatics computing for the genomics community
Source: BMC Bioinformatics. 2012 Mar 19;13:42. doi: 10.1186/1471-2105-13-42 (PMC3372431; doi:10.1186/1471-2105-13-42)
Supplement: Additional file 1 — Supplementary 1 Cloud BioLinux software documentation in the form of a mini, self-contained website. Users need to download and uncompress the .zip file, and open through a web browser the "index.html" file available on the main directory. (ZIP 1823 kb). [file 1471-2105-13-42-S1.ZIP › Cloud-BioLinux-Package-Documentation/docs/estscan.html]

Bio-Linux Software Documentation Pages

Back to search form

## estscan

|  |  |
| --- | --- |
| Name | estscan |
| Description | **ESTscan** is a program that can detect coding regions in DNA sequences, even if they are of low quality. It will also detect and correct sequencing errors that lead to frameshifts.  Similarly to genscan, ESTscan uses a Markov model to represent the bias in hexanucleotide usage found in coding regions relative to non-coding regions. Additionally, ESTscan allows insertions and deletions when these improve the coding region statistics.  **References:**  Lottaz C, Iseli C, Jongeneel CV, Bucher P. (2003) Modeling sequencing errors by combining Hidden Markov models Bioinformatics 19, ii 103-ii112. Iseli C, Jongeneel CV, Bucher P. (1999) ESTScan: a program for detecting, evaluating, and reconstructing potential coding regions in EST sequences. Proc Int Conf Intell Syst Mol Biol.138-48. |
| Homepage | http://www.ch.embnet.org/software/ESTScan2.html |
| Remote Documentation | http://www.ch.embnet.org/software/ESTScan2\_help.html |
